# Crystal structure of 4-formyl­pyridine semicarbazone hemihydrate

**DOI:** 10.1107/S2056989015007276

**Published:** 2015-04-18

**Authors:** Mayara Hissami Inoue, Davi F. Back, Robert A. Burrow, Fábio Souza Nunes

**Affiliations:** a Universidade Federal do Paraná, Departamento de Química, C.P. 19081, CEP 81531-980, Curitiba – PR, Brazil; b Universidade Federal do Santa Maria, Departamento de Química, CEP 97105-900, Santa Maria – RS, Brazil

**Keywords:** crystal structure, 4-formyl­pyridine semicarbazone hemihydrate, N—H⋯O hydrogen bonds

## Abstract

The mol­ecule of the title compound C_7_H_8_N_4_O·0.5H_2_O, alternatively called (*E*)-1-(pyridin-4-yl­methyl­ene)semi­carb­azide hemihydrate, is in the *E* conformation and is almost planar; the r.m.s. deviation of the positions of the atoms of the pyridine ring from the best-fit plane is 0.0039 Å. The C, N and O atoms of the rest of the mol­ecule sits close on this plane with a largest deviation of 0.115 (4) Å for the O atom of the semicarbazone moiety. There is an intra­molecular N—H⋯N hydrogen bond. In the crystal, mol­ecules are linked into an infinite three-dimensional network by classical N—H⋯O_s_ (s = semicarbazone) and O_w_—H⋯N (w = water) hydrogen bonds.

## Related literature   

For the preparation of coordination compounds of 4-formyl­pyridine semicarbazone with cobalt and zinc, see: Zhou *et al.* (2006*a* ▸,*b* ▸). For the spectroscopic (FT–IR, NMR and UV–vis) properties of 4- and 3-formyl­pyridine semicarbazones, see: Beraldo *et al.* (2001 ▸). For the crystal structure of 4-formyl­pyridine thio­semicarbazone, see: Restivo & Palenik (1970 ▸). For the crystal structures of 2-formyl­pyridine semicarbazone and several coordination compounds published by our group, see: Garbelini *et al.* (2008 ▸, 2009 ▸, 2011 ▸, 2012 ▸). Geometrical analysis was performed with *Mogul* (Bruno *et al.*, 2004 ▸).
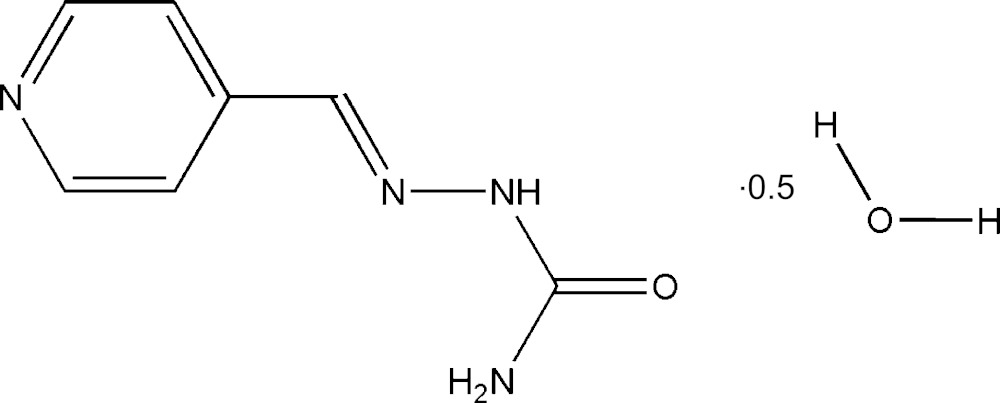


## Experimental   

### Crystal data  

2C_7_H_8_N_4_O·H_2_O
*M* *_r_* = 346.36
Monoclinic, 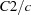

*a* = 25.0636 (13) Å
*b* = 5.3725 (3) Å
*c* = 13.0124 (7) Å
β = 111.717 (3)°
*V* = 1627.81 (16) Å^3^
*Z* = 4
Mo *K*α radiation
μ = 0.11 mm^−1^
*T* = 296 K
0.82 × 0.27 × 0.20 mm

### Data collection  

Bruker X8 Kappa APEXII diffractometer
Absorption correction: multi-scan (*SADABS*; Bruker, 2014 ▸) *T*_min_ = 0.860, *T*_max_ = 0.937
20739 measured reflections
1795 independent reflections
1502 reflections with *I* > 2σ(*I*)
*R*_int_ = 0.029

### Refinement  

*R*[*F* ^2^ > 2σ(*F* ^2^)] = 0.034
*wR*(*F* ^2^) = 0.094
*S* = 1.04
1795 reflections
127 parameters
1 restraint
H atoms treated by a mixture of independent and constrained refinement
Δρ_max_ = 0.23 e Å^−3^
Δρ_min_ = −0.20 e Å^−3^

### 

Data collection: *APEX2* (Bruker, 2014 ▸); cell refinement: *SAINT* (Bruker, 2014 ▸); data reduction: *SAINT*; program(s) used to solve structure: *SHELXT2014* (Sheldrick, 2015 ▸ ▸); program(s) used to refine structure: *SHELXL2014* (Sheldrick, 2015 ▸ ▸); molecular graphics: *DIAMOND* (Crystal Impact, 2014 ▸); software used to prepare material for publication: *SHELXL2014*.

## Supplementary Material

Crystal structure: contains datablock(s) global, I. DOI: 10.1107/S2056989015007276/lr2134sup1.cif

Structure factors: contains datablock(s) I. DOI: 10.1107/S2056989015007276/lr2134Isup2.hkl

Supporting information file. DOI: 10.1107/S2056989015007276/lr2134Isup3.cml

**Fig. 1.**
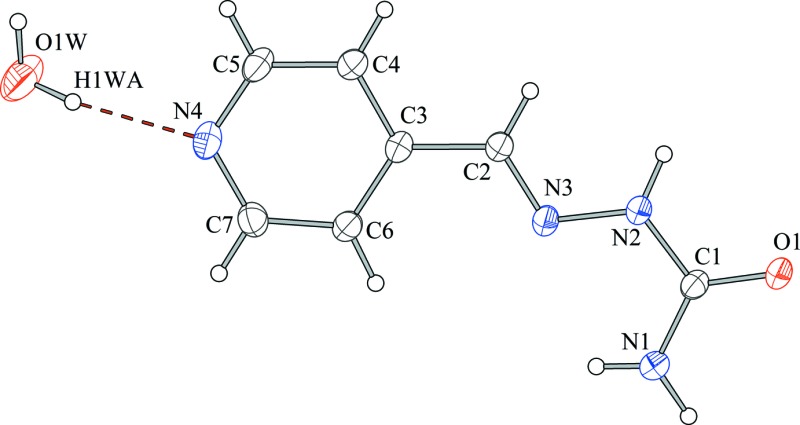
View of the title molecule.Displacement ellipsoids are drawn at the 50% probability level.

**Fig. 2.**
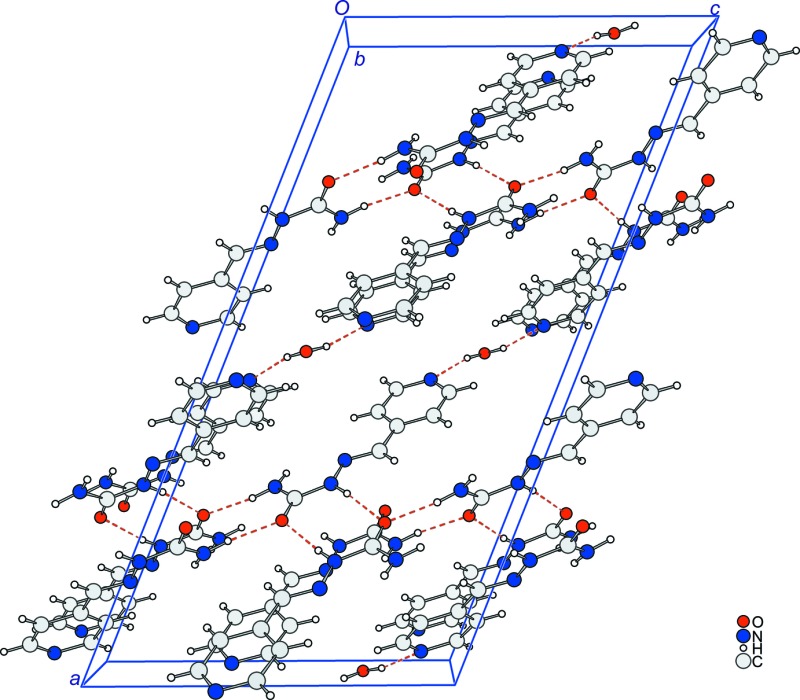
The molecular packing viewed down the crystallograpic b axis, with the a axis pointed down, showing the three dimensional hydrogen bonding network.

. DOI: 10.1107/S2056989015007276/lr2134fig1.tif
View of the title mol­ecule·Displacement ellipsoids are drawn at the 50% probability level.

b a . DOI: 10.1107/S2056989015007276/lr2134fig2.tif
The mol­ecular packing viewed down the crystallograpic *b* axis, with the *a* axis pointed down, showing the three dimensional hydrogen bonding network.

CCDC reference: 1059160

Additional supporting information:  crystallographic information; 3D view; checkCIF report

## Figures and Tables

**Table 1:** Hydrogen-bond geometry (, )

*D*H*A*	*D*H	H*A*	*D* *A*	*D*H*A*
N1H1*NA*N3	0.852(18)	2.273(18)	2.6541(16)	107.3(13)
N1H1*NB*O1^i^	0.917(19)	1.998(18)	2.9046(15)	169.3(16)
N2H2*N*O1^ii^	0.898(17)	2.022(17)	2.9141(14)	171.9(16)
O1*W*H1*WA*N4	0.87(3)	2.08(3)	2.9373(16)	168(3)

Symmetry codes: (i) 
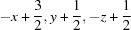
; (ii) 
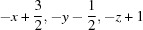
.

## supplementary crystallographic information

### S1. Structural commentary

The title molecule crystallizes in the E conformation and is almost ideally planar. The root mean square deviation of the postions of the atoms of the pyridine ring (C3/C4/C5/C6/C7/N4) from the best fit plane is 0.0039 Å. The C, N & O atoms of the rest of the molecule sits close on this plane with the largest deviation of 0.115 (4) Å for O1. The torsion angles of the side arm, C1—N2—N3—C2 (-174.35 (11)°, N2—N3—C2—C3 (-177.87 (10)° and N3—C2—C3—C6 (-1.76 (18)°) reflect this planarity. The shorter bond distance between C2 and N3 of 1.2752 (15) Å compared with 1.3366 (18) Å of N2—C1 along with the double bond character of C1=O1 at 1.2399 (14) Å is in accordance with the carbonyl tautomeric form. The longer than normal double bond length for N2—N3, 1.3706 (13) Å, and shorter than normal single bond length for C2—C3, 1.4616 (15) Å, indicates a resonance of the side chain with the pyridine ring.

There is an extensive three dimensional network of hydrogen bonding inter­actions formed between the 4-formyl­pyridine semicarbazone molecules, N2—H2N···O1 = 2.022 (18)Å and N1—H1NB···O1 = 1.998 (19)Å, and between the pyridine N4 atoms of the 4-formyl­pyridine semicarbazone molecules and the water molecules, O1W—H1WA···N4 = 2.077 (18)Å.

### S2. Database survey

An analysis of the geometry of the 4-formyl­pyridine semicarbazone molecule with Mogul (Bruno *et al.*, 2004) showed all bond lengths, bond angles, dihedral angles and ring geometry as not unusual, with the largest |z-score| = 1.254 for the C6–C3–C2 angle.

### S3. Synthesis and crystallization

Reagent grade chemicals were used in this work. The compound was prepared based on a similar reaction for the 2-formyl­pyridine semicarbazone (Garbelini *et al.* 2009).

In an round bottom flask 2.4 g (21 mmol) of semicarbazide hydro­chloride were dissolved in 20 mL of ethanol and 5 mL of water and mixed with 1.7 g (21 mmol) of sodium acetate and 15 mL of water. The mixture was kept under stirring and heating at 70°C until the complete dissolution. Then, 2.0 mL (21 mmol) of 4-formyl­pyridine was added and the resulting solution was cooled at -15 °C overnight. Colourless crystals were collected by filtration, washed with water and cold ethanol and dried under vacuum. The yield was 2.3 g (67%).

### S4. Refinement

Crystal data, data collection and structure refinement details are summarized in Table 1. Crystal data, data collection and structure refinement details are summarized in Table 1. Crystal data, data collection and structure refinement details are summarized in Table 1. The final structure was refined with SHELXL2014 (Sheldrick, 2015) with anisotropic displacement parameters for all non-hydrogen atoms; hydrogen atoms were refined isotropically as riding atoms at their theoretical ideal positions. Drawings were made with Crystal Impact Diamond 3.

### Crystal data

**Table:**

2C_7_H_8_N_4_O·H_2_O	*F*(000) = 728
*M**_r_* = 346.36	*D*_x_ = 1.413 Mg m^−^^3^
Monoclinic, *C*2/*c*	Mo *K*α radiation, λ = 0.71073 Å
*a* = 25.0636 (13) Å	Cell parameters from 5941 reflections
*b* = 5.3725 (3) Å	θ = 2.8–26.2°
*c* = 13.0124 (7) Å	µ = 0.11 mm^−^^1^
β = 111.717 (3)°	*T* = 296 K
*V* = 1627.81 (16) Å^3^	Block, clear light colourless
*Z* = 4	0.82 × 0.27 × 0.20 mm

### Data collection

**Table:**

Bruker X8 Kappa APEXII diffractometer	1795 independent reflections
Radiation source: sealed ceramic X ray tube, Siemens KFF	1502 reflections with *I* > 2σ(*I*)
Graphite crystal monochromator	*R*_int_ = 0.029
Detector resolution: 8.3333 pixels mm^-1^	θ_max_ = 27.1°, θ_min_ = 1.8°
0.5 ° ω & φ scans	*h* = −32→32
Absorption correction: multi-scan (*SADABS*; Bruker, 2014)	*k* = −6→6
*T*_min_ = 0.860, *T*_max_ = 0.937	*l* = −16→16
20739 measured reflections	

### Refinement

**Table:**

Refinement on *F*^2^	Secondary atom site location: difference Fourier map
Least-squares matrix: full	Hydrogen site location: mixed
*R*[*F*^2^ > 2σ(*F*^2^)] = 0.034	H atoms treated by a mixture of independent and constrained refinement
*wR*(*F*^2^) = 0.094	*w* = 1/[σ^2^(*F*_o_^2^) + (0.0436*P*)^2^ + 0.8364*P*] where *P* = (*F*_o_^2^ + 2*F*_c_^2^)/3
*S* = 1.04	(Δ/σ)_max_ = 0.001
1795 reflections	Δρ_max_ = 0.23 e Å^−^^3^
127 parameters	Δρ_min_ = −0.20 e Å^−^^3^
1 restraint	Extinction correction: *SHELXL*
Primary atom site location: structure-invariant direct methods	Extinction coefficient: 0.0067 (8)

### Special details

**Table:**

Geometry. All e.s.d.'s (except the e.s.d. in the dihedral angle between two l.s. planes) are estimated using the full covariance matrix. The cell e.s.d.'s are taken into account individually in the estimation of e.s.d.'s in distances, angles and torsion angles; correlations between e.s.d.'s in cell parameters are only used when they are defined by crystal symmetry. An approximate (isotropic) treatment of cell e.s.d.'s is used for estimating e.s.d.'s involving l.s. planes.

### Fractional atomic coordinates and isotropic or equivalent isotropic displacement parameters (Å^2^)

**Table:**

	*x*	*y*	*z*	*U*_iso_*/*U*_eq_	
O1	0.74966 (4)	−0.16017 (16)	0.36432 (7)	0.0411 (2)	
N1	0.70517 (5)	0.2096 (2)	0.30449 (9)	0.0410 (3)	
H1NA	0.6859 (8)	0.326 (3)	0.3188 (14)	0.061*	
H1NB	0.7226 (7)	0.234 (3)	0.2545 (15)	0.061*	
N2	0.69806 (5)	0.00971 (19)	0.45499 (9)	0.0373 (3)	
H2N	0.7110 (7)	−0.107 (3)	0.5079 (14)	0.056*	
N3	0.66491 (4)	0.20246 (18)	0.46659 (8)	0.0336 (3)	
N4	0.54246 (5)	0.7238 (2)	0.61921 (10)	0.0430 (3)	
C1	0.71939 (5)	0.0154 (2)	0.37260 (9)	0.0315 (3)	
C2	0.65096 (5)	0.1908 (2)	0.55129 (10)	0.0329 (3)	
H2	0.6646	0.0596	0.6007	0.039*	
C3	0.61410 (5)	0.3783 (2)	0.57272 (9)	0.0309 (3)	
C4	0.59797 (5)	0.3495 (2)	0.66315 (10)	0.0374 (3)	
H4	0.6108	0.2134	0.71	0.045*	
C5	0.56274 (6)	0.5251 (3)	0.68271 (11)	0.0431 (3)	
H5	0.5526	0.5034	0.744	0.052*	
C6	0.59294 (5)	0.5840 (2)	0.50545 (10)	0.0369 (3)	
H6	0.6025	0.6106	0.4437	0.044*	
C7	0.55757 (6)	0.7483 (2)	0.53140 (11)	0.0411 (3)	
H7	0.5434	0.8843	0.4851	0.049*	
O1W	0.5	1.0598 (3)	0.75	0.1046 (9)	
H1WA	0.5078 (14)	0.965 (5)	0.703 (2)	0.157*	

### Atomic displacement parameters (Å^2^)

**Table:**

	*U*^11^	*U*^22^	*U*^33^	*U*^12^	*U*^13^	*U*^23^
O1	0.0538 (6)	0.0380 (5)	0.0420 (5)	0.0108 (4)	0.0303 (4)	0.0010 (4)
N1	0.0519 (7)	0.0410 (6)	0.0391 (6)	0.0099 (5)	0.0274 (5)	0.0071 (5)
N2	0.0484 (6)	0.0338 (5)	0.0396 (6)	0.0118 (5)	0.0278 (5)	0.0057 (4)
N3	0.0372 (5)	0.0318 (5)	0.0370 (5)	0.0045 (4)	0.0198 (4)	−0.0008 (4)
N4	0.0427 (6)	0.0411 (6)	0.0521 (7)	0.0027 (5)	0.0257 (5)	−0.0070 (5)
C1	0.0351 (6)	0.0328 (6)	0.0304 (6)	−0.0009 (5)	0.0164 (5)	−0.0032 (4)
C2	0.0353 (6)	0.0327 (6)	0.0349 (6)	0.0026 (5)	0.0178 (5)	0.0007 (4)
C3	0.0304 (6)	0.0318 (6)	0.0334 (6)	−0.0030 (4)	0.0150 (5)	−0.0058 (4)
C4	0.0397 (7)	0.0393 (6)	0.0385 (6)	0.0015 (5)	0.0208 (5)	0.0015 (5)
C5	0.0462 (7)	0.0492 (7)	0.0443 (7)	−0.0004 (6)	0.0289 (6)	−0.0046 (6)
C6	0.0427 (7)	0.0369 (6)	0.0363 (6)	0.0018 (5)	0.0206 (5)	−0.0003 (5)
C7	0.0435 (7)	0.0339 (6)	0.0472 (7)	0.0041 (5)	0.0185 (6)	0.0000 (5)
O1W	0.200 (3)	0.0427 (9)	0.1390 (19)	0	0.142 (2)	0

### Geometric parameters (Å, º)

**Table:**

O1—C1	1.2399 (14)	C2—H2	0.93
N1—C1	1.3294 (16)	C3—C4	1.3870 (16)
N1—H1NA	0.854 (18)	C3—C6	1.3875 (17)
N1—H1NB	0.917 (19)	C4—C5	1.3789 (17)
N2—C1	1.3639 (15)	C4—H4	0.93
N2—N3	1.3706 (13)	C5—H5	0.93
N2—H2N	0.899 (17)	C6—C7	1.3789 (17)
N3—C2	1.2752 (15)	C6—H6	0.93
N4—C5	1.3301 (18)	C7—H7	0.93
N4—C7	1.3366 (18)	O1W—H1WA	0.874 (17)
C2—C3	1.4616 (15)		
			
C1—N1—H1NA	117.8 (12)	C4—C3—C2	119.22 (11)
C1—N1—H1NB	120.2 (10)	C6—C3—C2	123.34 (10)
H1NA—N1—H1NB	120.5 (16)	C5—C4—C3	119.20 (12)
C1—N2—N3	119.97 (10)	C5—C4—H4	120.4
C1—N2—H2N	118.8 (11)	C3—C4—H4	120.4
N3—N2—H2N	120.3 (11)	N4—C5—C4	123.92 (12)
C2—N3—N2	115.43 (10)	N4—C5—H5	118.0
C5—N4—C7	116.50 (11)	C4—C5—H5	118.0
O1—C1—N1	124.12 (11)	C7—C6—C3	119.07 (11)
O1—C1—N2	118.79 (10)	C7—C6—H6	120.5
N1—C1—N2	117.08 (10)	C3—C6—H6	120.5
N3—C2—C3	121.72 (11)	N4—C7—C6	123.87 (12)
N3—C2—H2	119.1	N4—C7—H7	118.1
C3—C2—H2	119.1	C6—C7—H7	118.1
C4—C3—C6	117.43 (11)		
			
C1—N2—N3—C2	−174.35 (11)	C2—C3—C4—C5	−179.38 (11)
N3—N2—C1—O1	179.42 (10)	C7—N4—C5—C4	0.5 (2)
N3—N2—C1—N1	−1.82 (17)	C3—C4—C5—N4	0.4 (2)
N2—N3—C2—C3	−177.87 (10)	C4—C3—C6—C7	0.24 (17)
N3—C2—C3—C4	176.79 (11)	C2—C3—C6—C7	178.81 (11)
N3—C2—C3—C6	−1.76 (18)	C5—N4—C7—C6	−1.1 (2)
C6—C3—C4—C5	−0.74 (18)	C3—C6—C7—N4	0.7 (2)

### Hydrogen-bond geometry (Å, º)

**Table:**

*D*—H···*A*	*D*—H	H···*A*	*D*···*A*	*D*—H···*A*
N1—H1*NA*···N3	0.852 (18)	2.273 (18)	2.6541 (16)	107.3 (13)
N1—H1*NB*···O1^i^	0.917 (19)	1.998 (18)	2.9046 (15)	169.3 (16)
N2—H2*N*···O1^ii^	0.898 (17)	2.022 (17)	2.9141 (14)	171.9 (16)
O1*W*—H1*WA*···N4	0.87 (3)	2.08 (3)	2.9373 (16)	168 (3)

Symmetry codes: (i) −*x*+3/2, *y*+1/2, −*z*+1/2; (ii) −*x*+3/2, −*y*−1/2, −*z*+1.
