# Supplementary figures and images for: Crystal structure of 4-formyl­pyridine semicarbazone hemihydrate
Source: Acta Crystallogr E Crystallogr Commun. 2015 Apr 18;71(Pt 5):o317–8. doi: 10.1107/S2056989015007276 (PMC4420042; doi:10.1107/S2056989015007276)

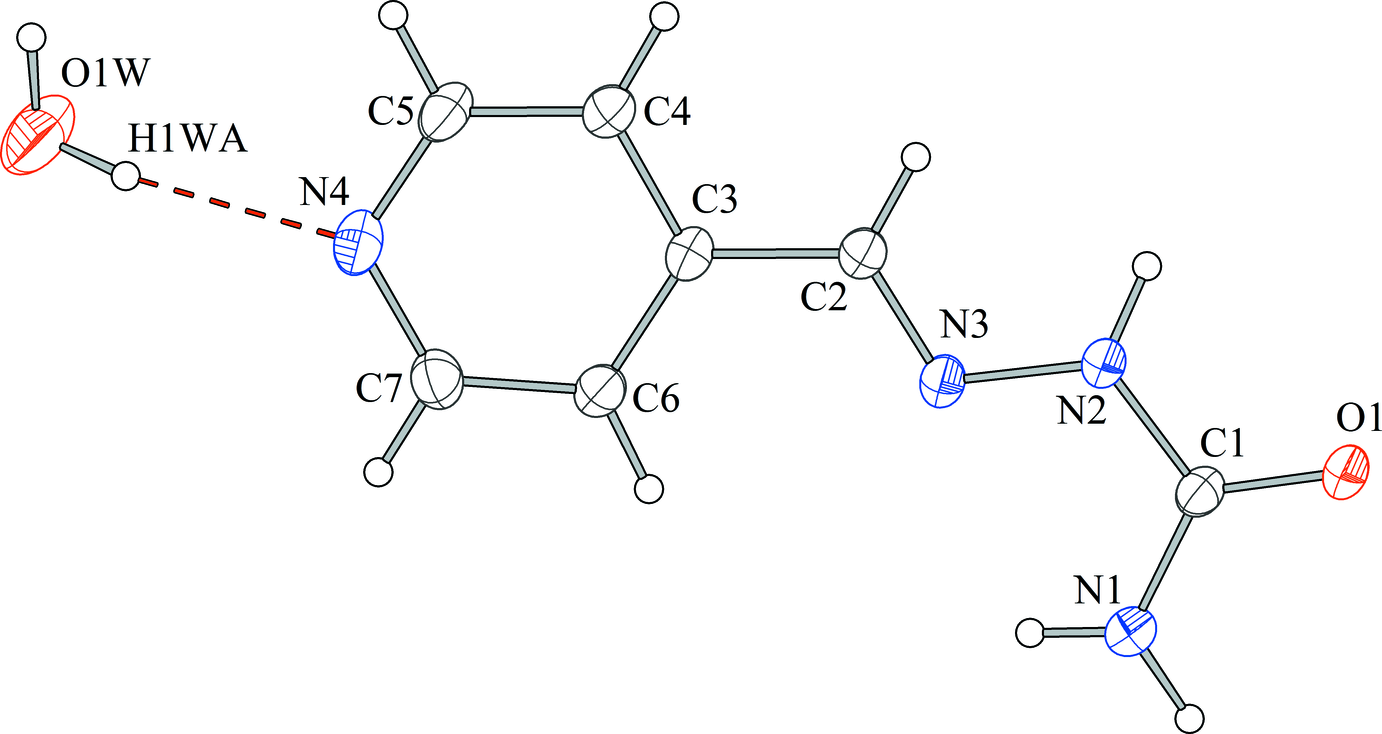

Supplement: Supplementary file 4 [file e-71-0o317-fig1.tif]

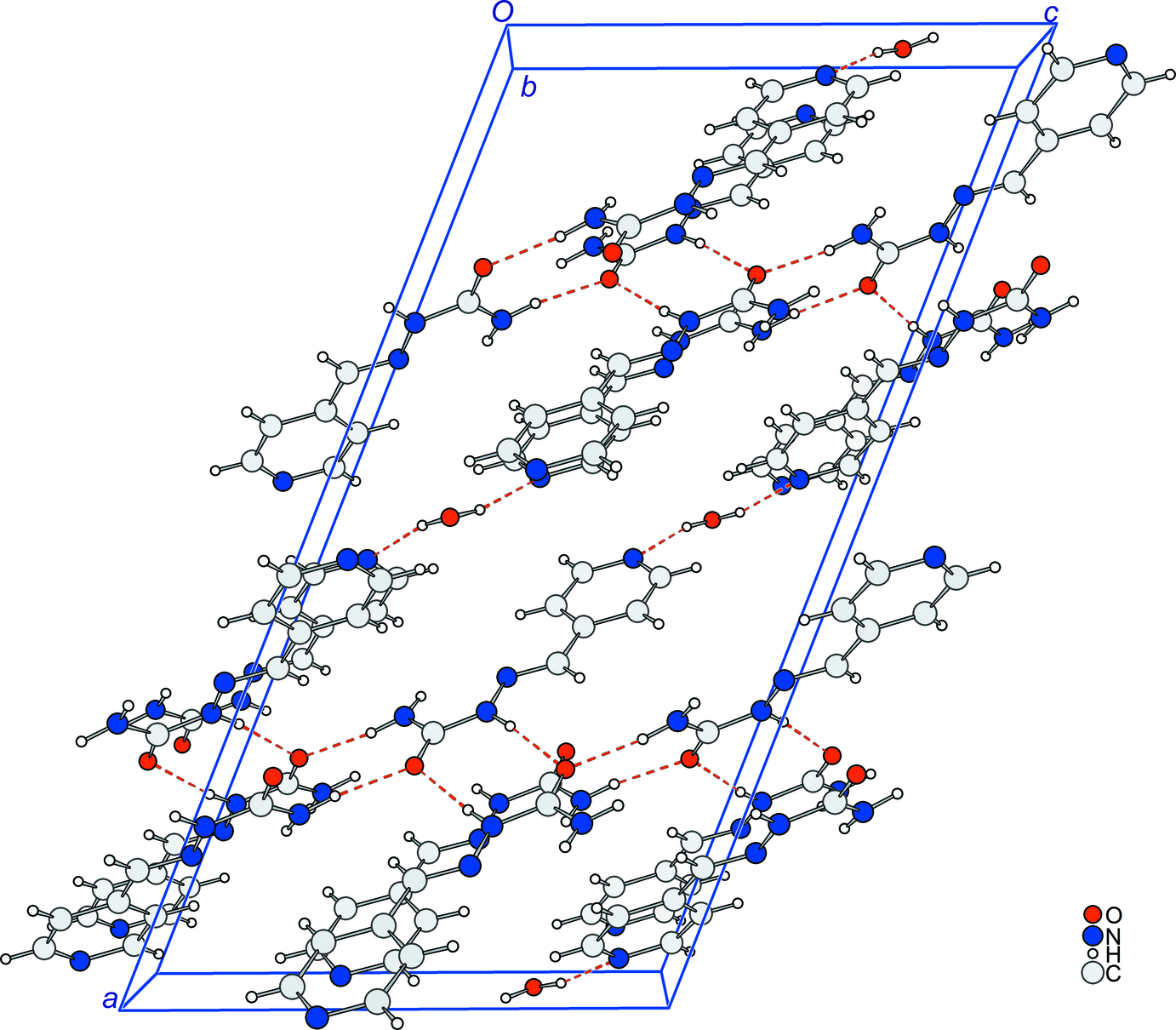

Supplement: Supplementary file 5 [file e-71-0o317-fig2.tif]
